# The Relationship of HbA1c Serum Levels with the Severity of Periodontal Disease in Patients with Type 1 Diabetes Mellitus: A Cross-Sectional Study

**DOI:** 10.1055/s-0044-1795123

**Published:** 2024-12-30

**Authors:** Rosana Costa, Marco Infante da Câmara, Fernando Figueira, José Júlio Pacheco, Catarina Pereira, Maria Gonçalves, Marta Relvas

**Affiliations:** 1 Department of Medicine and Oral Surgery, University Institute of Health Sciences (IUCS-CESPU), Gandra, Portugal; 2 Oral Pathology and Rehabilitation Research Unit (UNIPRO), University Institute of Health Sciences (IUCS-CESPU), Gandra, Portugal; 3 Department of Stomatology, Hospitalar Center of Tâmega e Sousa, Penafiel, Portugal; 4 Department of Endocrinology, Hospitalar Center of Tâmega e Sousa, Penafiel, Portugal; 5 Associate Laboratory i4HB-Institute for Health and Bioeconomy, University Institute of Health Sciences-CESPU, Gandra, Portugal; 6 UCIBIO-Applied Molecular Biosciences Unit, Translational Toxicology Research Laboratory, University Institute of Health Sciences (1H-TOXRUN, IUCS-CESPU), Gandra, Portugal

**Keywords:** glycated hemoglobin, type 1 diabetes mellitus, periodontitis, oral hygiene habits

## Abstract

**Objective** According to the evidence, the level of glycemic control is of key importance in determining the increased risk of periodontal disease (PD). The aim of the study was to evaluate the role of metabolic control as a key factor leading to the development and severity of periodontitis and compare the periodontal and oral hygiene status with the glycated hemoglobin levels.

**Materials and Methods** The evaluation was undertaken with diabetic patients (59 uncontrolled diabetics and 36 controlled diabetics) from a patient cohort of the Hospitalar Center of Tâmega e Sousa and subjects without diabetes ( *n* = 95).

**Statistical Analysis** The data were analyzed using IBM SPSS Statistics software (Statistical Program for Social Sciences), version 29.0 for Windows. In the logistic regression analysis, odds ratios (ORs) and 95% confidence intervals (CIs) were calculated. The significance level was set at 0.05.

**Results** Periodontal parameters were increased in systemically compromised individuals especially those who were poorly controlled as compared with their healthy counterparts, which are important indicators of PD progression. Furthermore, uncontrolled type 1 diabetic mellitus (T1DM) patients showed increased plaque index (PI), which predisposes these individuals to a greater degree of periodontal destruction and tooth loss. Using a binary logistic regression, we observe a significant relation of the risk of severe periodontitis in T1DM subjects with family history of T1DM ( *p* = 0.019; OR: 3.36; 95% CI: 1.22–9.21), alcohol consumption ( *p* = 0.02; OR: 3.78; 95% CI: 1.23–11.63), periodontal probing depth (PPD) ( *p* < 0.001; OR: 3.64; 95% CI: 14.74–90.34), and clinical attachment loss ( *p* = 0.033; OR: 4.71; 95% CI: 1.13–19.59).

**Conclusion** Increased glycated hemoglobin levels were positively related with periodontal status. Uncontrolled systemically compromised individuals showed an increased Plaque index (PI), which predisposes to greater periodontal inflammation and tooth loss. Increased clinical attachment level, Periodontal probing depth (PPD) and family history of T1DM, and alcohol consumption were identified as potential risk factors for severe periodontitis in subjects with T1DM.

## Introduction

Type 1 diabetes mellitus (T1DM) is an organ-specific autoimmune disease leading to abnormal fat, carbohydrate, and protein metabolism due to the absence of insulin. 1 2 3 The onset of the disease is usually made at a young age; however, it can also appear in adults and elderly individuals. 3

Periodontal disease (PD) is a chronic inflammatory disease caused by the presence of specific anaerobic pathogens contained in the subgingival biofilm. 4 5 6 PD not only affects oral health but also systemic health. 7 The development and progression of PD are directly linked to oral hygiene, and maintaining good oral hygiene reduces the risk of periodontitis. 8 9 10 Poor oral hygiene increases the risk of periodontitis by two to five times. 11

According to the literature, there is a relationship between diabetes and PD, with PD being the sixth complication of diabetes. 1 12 13 14 The relationship between diabetes and periodontitis is symbiotic because persistent hyperglycemia has been shown to negatively influence oral health, and severe periodontitis can have a negative impact on both glycemic control and diabetic complications. 15 Among patients with diabetes, a poor metabolic control were associated with a higher prevalence and severity of PD. 4 Consequently, the progressive destruction of connective tissue attachment and bone support, that occur in PD, can lead in tooth loss. 4 5 16 Clinical attachment loss (CAL) and periodontal probing depth (PPD) are metrics used to quantify the degree of tissue degradation of the tooth's supporting tissues. In addition, bleeding on probing (BOP) has been associated with a higher risk for attachment loss. 17 Several population-based studies have demonstrated that adults with diabetes are at higher risk of tooth loss than nondiabetic patients, especially those with poor metabolic control. 16 18 19 The glycosylated hemoglobin (HbA1c) level provides a dependable index for measurement of glycemic control in diabetic patients. 20 The average of blood glucose concentration in diabetic individuals, for the last 2 to 3 months, is provided with this test, with a lower HbA1c (<7%) indicating better metabolic control. 20 21

The mechanism by which diabetes may influence PD includes vascular abnormalities, neutrophil dysfunction, anomalies in collagen synthesis, and genetic predisposition. 16 22 Diabetes Mellitus (DM) impairs the activity of immune system cells such as, neutrophils, macrophages, and monocytes. Thus, neutrophil adhesion, chemotaxis, and phagocytosis are weakened, resulting in decreased bacterial mortality and increased periodontal damage. 23 24 Diabetes increases inflammatory mediators such as tumor necrosis factor-α (TNF-α) and interleukin-1β (IL-1β) in periodontal tissues, causing inflammation. This increase in proinflammatory cytokines is responsible for the chronic process of atheromatous lesion formation in large vessels. 24 25

Besides being associated with various diseases, smoking is a major risk factor for the development of periodontitis. The effect of smoking on periodontal tissues has been discussed for decades, and it has been shown that smokers have more periodontal problems than nonsmokers. 1 19 Studies performed on smokers have revealed alterations in gingival crevicular fluid in relation to immune cell function, changes in the regulation of proteolytic synthesis, and altered inflammatory cytokine profiles. Smoking impacts the immune system (delays neutrophil recruitment and migration), the microbiota (subgingival biofilm composition changes with increased periodontal pathogen prevalence), and the periodontium's ability to heal (higher collagenolytic activity combined with fewer gingival blood vessels). 26 27 28 29 According to the World Health Organization, diabetic patients who smoke are 1.7 times more likely to develop PD than those who do not. 1

The aim of this study is to evaluate the role of metabolic control as a key factor leading to the development and severity of periodontitis and compare the periodontal and oral hygiene status with the HbA1c.

## Materials and Methods

### Study Design

This cross-sectional study was conducted in accordance with the Declaration of Helsinki approved by the Ethical Committee of the Hospitalar Center of Tâmega e Sousa, Penafiel (reference: 22/2022). All participants signed informed consent to undergo physical and periodontal examinations. The study complied with the STROBE guidelines. 30

Patients with an established diagnosis of T1DM according to the World Health Organization criteria 31 were consecutively recruited from among those who came for regular checkups at the Outpatient Diabetes Center, Penafiel (Portugal) from November 2022 to July 2023.

The following inclusion criteria were considered: (1) having at least eight teeth and (2) availability of measurements of routine diabetes laboratory tests made in 6 months before enrollment. Exclusion criteria were: (1) T2DM; (2) intake of drugs known to affect gingival tissues, use of antibiotics, steroidal, and/or nonsteroidal anti-inflammatory drugs 3 weeks prior to the visit; (3) periodontal therapy in the past 6 months; (4) pregnancy or lactation; and (5) diagnosis of following pathologies: cancer, human immunodeficiency virus/AIDS, chronic infections, liver/kidney failure excluding diabetic nephropathy, chronic obstructive pulmonary disease with acute episodes, and/or requiring the use of steroidal inhalator.

### Data Collection

The convenience sample was constructed with patients and controls and selected sequentially within a period of time.

Participants were required to complete a questionnaire to obtain information on sociodemographic characteristics (gender, age, education), general health behavior (daily smoking, alcohol consumption), and oral hygiene behavior (toothbrush frequency, use of interdental devices).

Data on medical history, family background of T1DM, T1DM onset and duration, cardiovascular risk factors, chronic T1DM complications, as well as results of laboratory tests performed in the last diabetic visit (HbA1c level, lipid profile, and creatinine) were collected.

Two masked diabetes specialists reviewed the medical history of the participants and conducted a physical examination including anthropometric measurements (weight, height, and waist circumference), palpation, and auscultation. The body mass index was calculated as weight/height squared (kg/m ^2^ ).

Subsequently, two periodontologists conduct a periodontal examination. To ensure inter- and intraexaminer reproducibility, measurements of periodontal parameters were repeated in 20% of the sample and compared with those recorded by a gold-standard examiner. The *k* coefficients (within 1 mm) between examiners ranged from 0.79 to 0.93 in the evaluation of PPD and from 0.81 to 0.89 in the evaluation of gingival recession (REC). The intraexaminer concordance rates for repeated measurements were 0.89 to 0.95 for PPD and 0.82 to 0.91 for REC.

Full-mouth PPD, REC, and CAL were recorded by means of a periodontal probe with 1-mm markings (PCP-UNC 15, Hu-Friedy, Chicago, Illinois, United States) at six sites per tooth, excluding third molars. The number of missing teeth was also recorded.

The presence of periodontitis was defined according to the criteria proposed by the current consensus report of the 2017 World Workshop on the Classification of Periodontal and Peri-Implant Diseases and Conditions. 32

### Statistical Analysis

The data were analyzed using IBM SPSS Statistics software (Statistical Program for Social Sciences), version 29.0 for Windows.

Descriptive statistics were expressed as means and standard deviations for quantitative variables and as frequencies and percentages for qualitative variables.

Categorical grouping variables included periodontal status (no periodontitis, periodontitis I/II, and periodontitis III/IV) and glycemic control (controlled: HbA1c < 7%; uncontrolled: HbA1c ≥ 7%). The education system was divided into four corresponding levels: second cycle (6 years of education), third cycle (9 years of education), secondary education (12 years of education), and college (higher education).

With regard to metabolic control, it is important to understand that the treatment of diabetes is personalized, so the analysis of the HbA1c value is always adjusted to each patient's profile, namely their comorbidities, age, lifestyle, and habits. However, for the purposes of statistical treatment of the data and for comparison with data from the same area of interest, we used the *American Diabetes Association* classification to assess HbA1c values regarding metabolic control. Thus, patients with a HbA1c value <7% were considered to tend to be metabolically controlled, and patients with a HbA1c value ≥7% were considered to tend to be metabolically uncontrolled. 21

The Shapiro–Wilk's test was used to assess the normality of the sample, with no evidence of rejection of the null hypotheses. The normality of the data led to the adoption of a parametric analysis. The chi-square test/Fisher's exact test was used to compare nondiabetic, controlled diabetic, and uncontrolled diabetic individuals with other explanatory variables such as gender, age groups, smoking habits, oral hygiene habits, and periodontal status, as well as to compare sociodemographic and clinical variables in patients with T1DM according to periodontal status. To compare periodontal indices, the number of teeth and HbA1c values between the different groups, one-way analysis of variance (ANOVA) was used, followed by the Bonferroni's test. The effect sizes for the ANOVA were determined using η ^2^ values, with the thresholds considered being η ^2^ = 0.01 for a small effect, η ^2^ = 0.06 for a medium effect, and η ^2^ = 0.14 for a large effect. A binary logistic regression was performed to assess the relationship between severe periodontitis and the predictor variables. To select the best set of predictors, a stepwise variable selection technique was used. This approach combines forward and backward selection, iteratively adding or removing variables based on their statistical significance ( *p* < 0.05). The final model included the list of significant predictors, which were found to significantly influence the probability of severe periodontitis occurring. To assess potential multicollinearity among the predictor variables, variance inflation factors (VIFs) were calculated. Variables with a VIF greater than 5 were considered to exhibit high multicollinearity and were removed from the model to ensure robust estimates. The final model included the list of significant predictors, all of which were retained based on their statistical significance and lack of multicollinearity.

In the logistic regression analysis, odds ratios (ORs) and 95% confidence intervals (CIs) were calculated. The significance level was set at 0.05.

## Results

### Demographic Characteristics, Smoking Habits, and Oral Hygiene Habits

A total of 190 individuals voluntarily took part in the study, 95 nondiabetics (controls), 59 uncontrolled diabetics, and 36 controlled diabetics, 63.2% of whom were male. The demographic characteristics of the study groups, as well as their smoking and oral hygiene habits, are shown in Table 1 . With regard to age, we found that the majority of nondiabetics (46.3%) and noncontrolled diabetics (42.4%) were aged between 26 and 46 years, with 50% of controlled diabetics aged younger than 25 years. We observed a statistically significant relationship between schooling and group belonging (χ ^2^ [6] = 52.70; *p* < 0.001), with the lowest level of schooling being found in uncontrolled diabetics, where of the 59 individuals, 27 (45.7%) had either a second (28.8%) or third cycle degree (16.9%). As for smoking habits, we found a total of 150 (78.9%) nonsmokers and 40 (21.1%) active smokers, of whom 25.4% were uncontrolled diabetics, 22.1% nondiabetics, and 11.1% controlled diabetics; 53.3% of uncontrolled diabetics, 33.3% of nondiabetics, and 25.0% of controlled diabetics smoke more than 10 cigarettes a day.

**Table 1:** Association of diabetic and nondiabetic groups with different explanatory variables

	Nondiabetics ( *n* = 95)	Controlled T1DM ( *n* = 36)	Uncontrolled T1DM ( *n* = 59)	χ ^2^	*p* -Value
Age (y)					
≤ 25	32 (33.7)	18 (50.0)	15 (25.4)	8.1	0.09
26–45	44 (46.3)	11 (30.6)	25 (42.4)		
≥ 46	19 (20.0)	7 (19.4)	19 (32.2)		
Gender					
Male	60 (63.2)	23 (63.9)	39 (66.1)	0.14	0.93
Female	35 (36.8)	13 (36.1)	20 (33.9)		
Education level					
Second cycle a	0 (0.0)	2 (5.6)	17 (28.8)	52.7	< 0.001
Third cycle b	2 (2.1)	4 (11.1)	10 (16.9)		
Secondary c	68 (71.6)	17 (47.2)	21 (35.6)		
College d	25 (26.3)	13 (36.1)	11 (18.6)		
Active smoker					
Yes	21 (22.1)	4 (11.1)	15 (25.4)	2.9	0.24
No	74 (77.9)	32 (88.9)	44 (74.6)		
No. of cigarettes/d					
≤ 10 cigarettes	14 (66.7)	3 (75.0)	7 (46.7)	1.9	0.39
> 10 cigarettes	7 (33.3)	1 (25.0)	8 (53.3)		
Frequency of tooth brushing					
Once a day	0 (0.0)	5 (13.9)	16 (27.1)	39.1	< 0.001
Twice a day	45 (47.4)	19 (52.8)	34 (57.6)		
≥3 times a day	50 (52.6)	12 (33.3)	9 (15.3)		
Auxiliary means other than the brush					
Yes	45 (61.6)	14 (19.2)	14 (19.2)	8.6	0.014
No	50 (42.7)	22 (18.8)	45 (38.5)		

Abbreviation: T1DM, type 1 diabetic mellitus.

Note: Values indicate the number of subjects and percentages; *p* = level of significance; χ ^2^ =  chi-square test/Fisher's exact test.

a 6 years of education.

b 9 years of education.

c 12 years of education.

d Higher education.

Regarding oral hygiene habits, 57.6% of uncontrolled diabetics and 52.8% of controlled diabetics brush their teeth twice a day and 52.6% of non-diabetics brush three or more times a day (χ ^2^ [2] = 39.1; *p* < 0.001). Dental floss and/or brushes were used by 61.6% of nondiabetics and only 19.2% of controlled and noncontrolled diabetics, respectively, and this relationship was statistically significant (χ ^2^ [1] = 39.1; *p* = 0.014).

### Periodontal Status among Nondiabetics, Controlled Diabetics, and Uncontrolled Diabetics

As for the diagnosis of PD, there were statistically significant differences between the groups (χ ^2^ [4] = 61.4; *p* < 0.001), with periodontitis reaching a prevalence of 59.3% in uncontrolled diabetics, 44.4% in controlled diabetics, and 14.7% in nondiabetics. Gingivitis had the highest prevalence in controlled diabetics (36.1%), 33.9% in noncontrolled diabetics, and 21.1% in nondiabetics. Of the 59 uncontrolled diabetics, only 4 (6.8%) presented periodontal health ( Table 2 ). As for the extent of periodontitis, 82.9% of uncontrolled diabetics, 81.3% of controlled diabetics, and 50.0% of non-diabetics had generalized periodontitis (χ ^2^ [2] = 61.4; *p* = 0.045). There was a statistically significant relationship between the progression of periodontitis and the group of belonging (χ ^2^ [4] = 61.4; *p* < 0.001), with all the uncontrolled diabetics with periodontitis showing rapid progression (Grade C), 68.8% of the controlled diabetics showing moderate progression (Grade B) and 31.2% rapid progression, and 57.1% of nondiabetics showed slow progression (Grade A), 35.7% moderate progression and only one individual (7.1%) rapid progression (Grade C) ( Table 2 ).

**Table 2:** Association of diabetic and nondiabetic groups with periodontal status

	Nondiabetics ( *n* = 95)	Controlled T1DM ( *n* = 36)	Uncontrolled T1DM ( *n* = 59)	χ ^2^	*p* -Value
Periodontal status					
Periodontal health	61 (64.2)	7 (19.4)	4 (6.8)	61.4	< 0.001
Gingivitis	20 (21.1)	13 (36.1)	20 (33.9)		
Periodontitis	14 (14.7)	16 (44.4)	35 (59.3)		
Extent of periodontitis					
Generalized	7 (50.0)	13 (81.2)	29 (82.9)	6.2	0.045
Localized	7 (50.0)	3 (18.8)	6 (17.1)		
Periodontitis' grade					
Grade A	8 (57.1)	0 (0.0)	0 (0.0)	68.2	< 0.001
Grade B	5 (35.7)	11 (68.8)	0 (0.0)		
Grade C	1 (7.1)	5 (31.2)	35 (100.0)		

Abbreviation: T1DM, type 1 diabetic mellitus.

Note: Values indicate the number of subjects and percentages; *p* = level of significance; χ ^2^ = chi-square test/Fisher's exact test.

Table 3 shows the periodontal indices of nondiabetics, controlled diabetics, and uncontrolled diabetics. We observed statistically significant differences between nondiabetics and patients with controlled diabetes and uncontrolled diabetes in all periodontal conditions. In all cases, the plaque index (PI), (BOP, PPD (mm), and CAL (mm) were significantly higher in patients with uncontrolled diabetes compared with patients with controlled diabetes and nondiabetics ( *p* < 0.001).

**Table 3:** Comparative analysis of periodontal indices, number of teeth, and HbA1c with diabetic and ND groups

Variables	ND ( *n* = 95)	Controlled T1DM ( *n* = 36)	Uncontrolled T1DM ( *n* = 59)	*F*	Overall ( *p* -Value)	ND vs. CD ( *p* -Value)	ND vs. NCD ( *p* -Value)	CD vs. NCD ( *p* -Value)	η ^2^
PI	20.8 ± 18.8	38.2 ± 20.2	51.9 ± 30.9	32.9	< 0.001	< 0.001	< 0.001	0.019	0.2–0.4
BOP	10.1 ± 10.7	21.3 ± 18.9	31.41 ± 19.6	38.8	< 0.001	<0.001	< 0.001	0.641	0.2–0.4
PPD (mm)	1.8 ± 0.5	2.3 ± 0.5	2.7 ± 0.9	32.3	< 0.001	<0.001	< 0.001	0.02	0.2–0.3
CAL (mm)	1.1 ± 1.4	2.1 ± 1.8	2.6 ± 1.7	18.2	< 0.001	0.004	< 0.001	0.343	0.08–0.3
Teeth	28.3 ± 2.4	28.2 ± 3.1	24.9 ± 6.0	15.3	< 0.001	1.0	< 0.001	< 0.001	0.06–0.2
HbA1c	5.2 ± 0.3	6.3 ± 0.5	8.5 ± 1.1	410.6	< 0.001	< 0.001	< 0.001	< 0.001	0.8–0.9

Abbreviations: BOP, bleeding on probing; CAL,l clinical attachment loss; CD, controlled diabetes; HbA1c, glycosylated hemoglobin; NCD, noncontrolled diabetes; ND, nondiabetics; PI, plaque index; PPD, periodontal probing depth; T1DM, type 1 diabetic mellitus.

Note: η ^2^ = effect size; *p* = level of significance; *p* -value was derived from the one-way analysis of variance test and Bonferroni's test.

We observed that PI values were significantly higher in patients with uncontrolled diabetes (51.9 ± 30.9) compared with patients with controlled diabetes (PI = 38.2 ± 20.2) and nondiabetics (20.8 ± 18.8) ( *p* < 0.001; *p* < 0.001; *p* = 0.019). At the BOP level, there were statistically significant differences between nondiabetics (10.1 ± 10.7) and patients with controlled diabetes (21.3 ± 18.9) ( *p* < 0.001) and between controlled and uncontrolled diabetes (31.41 ± 19.6) ( *p* < 0.001). When analyzing the total PPD parameter (mm), we detected significantly higher values in patients with uncontrolled diabetes (2.7 ± 0.9 mm) compared with patients with controlled diabetes (2.3 ± 0.5) ( *p* = 0.02) and healthy controls ( *p* < 0.001). There were also statistically significant differences between the healthy controls and controlled diabetes groups ( *p* < 0.001). As for the total CAL parameter (mm), there were significantly higher values in patients with uncontrolled diabetes (2.6 ± 1.7) compared with nondiabetics (1.1 ± 1.4) ( *p* < 0.001) and controlled diabetics (2.1 ± 1.8) compared with nondiabetic patients ( *p* = 0.004). With regard to the number of teeth, nondiabetic patients (28.3 ± 2.4) had a higher average number of teeth compared with controlled diabetics (28.2 ± 3.1) and noncontrolled diabetics (24.9 ± 6.0), and these differences were statistically significant between patients with noncontrolled diabetes and patients with controlled diabetes (28.2 ± 3.1) ( *p* < 0.001) and between noncontrolled diabetics and nondiabetics ( *p* < 0.001). In terms of HbA1c, patients with uncontrolled diabetes showed significantly higher values (8.5 ± 1.1) compared with patients with controlled diabetes (6.3 ± 0.5) ( *p* < 0.001) and nondiabetics (5.2 ± 0.3) ( *p* < 0.001). There were also statistically significant differences between the nondiabetic and controlled diabetes groups ( *p* < 0.001).

When we analyzed diabetic patients according to their periodontal status ( Table 4 ) and the behavioral factors that modify or aggravate the disease, we found a statistically significant association between age and PD (χ ^2^ [4] = 25.9; *p* < 0.001), with the highest prevalence of periodontitis III/IV (60.9%) occurring in individuals older than 46 years. There was also a statistically significant association between schooling and the severity of periodontitis (χ ^2^ [6] = 23.4; *p* < 0.001), with 43.5% of patients with periodontitis III/IV having only completed secondary school (12 years of education). Overall, a statistically significant association was observed between periodontal condition and smoking habits (χ ^2^ [2] = 10.5; *p* = 0.05), and alcohol consumption (χ ^2^ [2] = 6.7; *p* = 0.036). Patients with periodontitis III/IV, compared with those with periodontitis I/II and periodontally healthy, had a higher frequency of family history of T1DM (56.5%) and complications (39.1%), with statistically significant differences ( *p* = 0.034; *p* = 0.044). Regarding the duration of diabetes, although this was not significantly related to periodontal condition, 52.2% of individuals with periodontitis III/IV had diabetes for more than 20 years and 26.1% between 11 and 20 years.

**Table 4:** Sociodemographic characteristics of T1DM patients according to the periodontal status

	Subject with healthy periodontium ( *n* = 44)	Patients with periodontal disease I/II ( *n* = 28)	Patients with periodontal disease III/IV ( *n* = 23)	χ ^2^	*p* -Value
Age (y)					
≤ 25	24 (54.5)	7 (25.0)	2 (8.7)	25.9	< 0.001
26–45	16 (36.4)	13 (46.4)	7 (30.4)		
≥ 46	4 (9.1)	8 (28.6)	14 (60.9)		
Gender					
Male	24 (54.5)	19 (67.9)	19 (82.6)	5.4	0.068
Female	20 (45.5)	9 (32.1)	4 (17.4)		
Education level					
Second cycle a	2 (4.5)	7 (25.0)	10 (43.5)	23.4	< 0.001
Third cycle b	4 (9.1)	4 (14.3)	6 (26.1)		
Secondary c	22 (50.0)	10 (35.7)	6 (26.1)		
College d	16 (36.4)	7 (25.0)	1 (4.3)		
Active smoker					
Yes	3 (6.8)	7 (25.0)	9 (39.1)	10.5	0.005
No	41 (93.2)	21 (75.0)	14 (60.9)		
No. of cigarettes/d					
≤ 10 cigarettes	3 (100.0)	4 (57.1)	5 (55.6)	3.2	0.201
> 10 cigarettes	0 (0.0)	3 (42.9)	4 (44.4)		
Alcohol consumption					
Yes	20 (45.5)	15 (53.6)	18 (78.3)	6.7	0.036
No	24 (54.5)	13 (13.7)	5 (5.3)		
Frequency of tooth brushing					
Once a day	7 (15.9)	8 (28.6)	6 (26.1)	4.8	0.310
Twice a day	25 (56.8)	13 (46.4)	15 (65.2)		
≥3 times a day	12 (27.3)	7 (25.0)	2 (8.7)		
Auxiliary means other than the brush					
Yes	15 (34.1)	7 (25.0)	6 (26.1)	0.8	0.655
No	29 (65.9)	21 (75.0)	17 (73.9)		
Family history of T1DM					
Yes	11 (25.0)	9 (32.1)	13 (56.5)	6.7	0.034
No	33 (75.0)	19 (67.9)	10 (43.5)		
Duration of diabetes (y)					
1–5	7 (15.9)	5 (17.9)	2 (8.7)	8.2	0.222
6–10	6 (13.6)	7 (25.0)	3 (13.0)		
11–20	20 (45.5)	9 (32.1)	6 (26.1)		
> 20	11 (25.0)	7 (25.0)	12 (52.2)		
Chronic complications of diabetes					
None	33 (75.0)	17 (60.7)	9 (39.1)	9.8	0.044
One	6 (13.6)	3 (10.7)	5 (21.7)		
Two or more	5 (11.4)	8 (28.6)	9 (39.1)		
Glycemic control					
Controlled	20 (45.5)	11 (39.3)	5 (21.7)	3.6	0.162
Uncontrolled	24 (54.5)	17 (60.7)	18 (78.3)		

Abbreviation: T1DM, type 1 diabetic mellitus.

Note: Values indicate the number of subjects and percentages; *p* = level of significance; χ ^2^ - = chi-square test/Fisher's exact test.

a 6 years of education.

b 9 years of education.

c 12 years of education.

d Higher education.

With regard to the clinical variables and as we can see in Table 5 , there were significantly higher levels of triglycerides (TG) in individuals with periodontitis III/IV (109.1 ± 75.3) compared with those with periodontitis I/II (83.0 ± 40.6) and periodontally healthy (76.6 ± 36.8) ( *p* = 0.041), these differences being established between the periodontitis III/IV and healthy groups ( *p* = 0.038). We observed that the percentage values of PI and BOP were higher in patients with periodontitis III/IV (PI, 63.5 ± 27.6; BOP, 40.8 ± 21.7) compared with patients with periodontitis I/II (PI, 35.9 ± 18.4; BOP, 35.9 ± 18.4) and periodontally healthy (PI, 37.9 ± 26.4; BOP, 20.3 ± 13.6) ( *p* = 0.001; *p* < 0.001). At the PI level, the differences reached statistical significance between the periodontitis III/IV and healthy groups ( *p* < 0.001). For BOP, the differences were between the healthy and periodontitis I/II groups ( *p* < 0.001) and between the healthy and periodontitis III/IV groups ( *p* < 0.001). When analyzing the PPD, we found significantly higher values in patients with periodontitis III/IV (3.6 ± 1.0) compared with those with periodontitis I/II (2.6 ± 0.4) ( *p* < 0.001) and periodontally healthy patients (2.0 ± 0.3) ( *p* < 0.001). In the CAL, it was patients with periodontitis III/IV who showed significantly higher values (3.8 ± 1.7) compared with patients with periodontitis I/II (2.4 ± 1.6) and healthy patients (1.6 ± 1.5). These differences reached statistical significance between healthy and periodontitis III/IV ( *p* < = 0.001), and between periodontitis I/II and periodontitis III/IV ( *p* < = 0.001). In addition, with regard to the number of teeth, there were statistically significant differences between the periodontally healthy and periodontitis III/IV groups ( *p* < 0.001) and between the periodontitis I/II and periodontitis III/IV groups ( *p* < 0.001).

**Table 5:** Clinical characteristics of T1DM patients according to the periodontal status

Variables	Subject with healthy periodontium ( *n* = 44)	Patients with periodontal disease I/II ( *n* = 28)	Patients with periodontal disease III/IV ( *n* = 23)	*F*	*p* -Value	η ^2^
Disease duration	2.8 ± 1.1 (0–4)	2.6 ± 1.2 (0–4)	3.2 ± 1.0 (1–4)	2.3	0.109	0–0.14
BMI (kg/m ^2^ )	25.5 ± 4.8 (18.2–45.7)	25.4 ± 3.5 (20.2–34.1)	25.5 ± 3.0 (19.2–30.2)	0.01	0.994	0.0–0.02
HbA1c	7.4 ± 1.4 (5.2–11.2)	7.6 ± 1.2 (5.2–10.4)	8.3 ± 1.6 (6.2–12.0)	2.8	0.066	0.0–0.16
TG (mg/dL)	76.6 ± 36.8 a (33.0–185.0)	83.0 ± 40.6 (37.0–190.0)	109.1 ± 75.3 a (31.0–389.0)	3.3	0.041	0.0–0.17
HDL-C (mg/dL)	56.9 ± 14.6 (39.0–102.0)	58.0 ± 18.2 (36.0–120.0)	56.4 ± 13.4 (35.0–120.0)	0.07	0.9	0.0–0.22
LDL-C (mg/dL)	92.6 ± 25.2 (34.0–145.0)	115.4 ± 131.4 (59.0–778.0)	89. 2 ± 31.4 (34.0–138.0)	1.0	0.362	0.01–0.02
Total cholesterol (mg/dL)	166.1 ± 37.9 (84.0–242.0)	165.5 ± 31.9 (106.0–228.0)	166.6 ± 34.8 (104.0–226.0)	0.01	0.994	0.0–0.02
Creatinine	0.9 ± 0.13 (0.7–1.3)	0.9 ± 0.19 (0.6–1.6)	1.5 ± 2.2 (0.7–11.0)	2.8	0.063	0.0–0.2
PI	37.9 ± 26.4 b	46.9 ± 25.2	63.5 ± 27.6 b	7.1	0.001	0.02–0.3
BOP	20.3 ± 13.6 c	35.9 ± 18.4 c	40.8 ± 21.7 c	13.0	< 0.001	0.08–0.13
PPD (mm)	2.0 ± 0.3 d	2.6 ± 0.4 d	3.6 ± 1.0 d	66.5	< 0.001	0.5–0.7
CAL (mm)	1.6 ± 1.5 e	2.4 ± 1.6 e	3.8 ± 1.7 e	20.6	< 0.001	0.2–0.4
Teeth	28.2 ± 2.7 f	26.7 ± 3.8 f	21.4 ± 7.5 f	16.3	< 0.001	0.1–0.4

Abbreviations: BMI, body mass index; BOP, bleeding on probing; CAL, clinical attachment loss; HbA1c, glycated hemoglobin; HDL-C, high-density lipoprotein cholesterol; LDL-C, low-density lipoprotein cholesterol; TG, triglycerides; PI, plaque index; PPD, periodontal probing depth.

Notes: η ^2^ = effect size. The *p* -value was derived from the one-way analysis of variance test and Bonferroni's test. *p* = level of significance.

a Statistically significant differences between healthy and periodontitis III/IV ( *p* = 0.038).

b Statistically significant differences between healthy and periodontitis III/IV *(p* < 0.001).

c Statistically significant differences between healthy and periodontitis III/IV ( *p* ≤ 0.001), and healthy and periodontitis I/II ( *p* < = 0.001).

d Statistically significant differences between healthy and periodontitis III/IV ( *p* ≤ 0.001), between healthy and periodontitis I/II ( *p* ≤ 0.001), and periodontitis III/IV and periodontitis I/II ( *p* ≤ 0.001).

e Statistically significant differences between healthy and periodontitis III/IV ( *p* < = 0.001), and between periodontitis I/II and periodontitis III/IV ( *p* < = 0.001).

f Statistically significant differences between periodontitis III/IV and healthy ( *p* < 0.001), and between periodontitis I/II and periodontitis III/IV ( *p* < 0.001).

The binary logistic regression models for severe periodontitis III/IV are shown in Table 6 . Model 1 showed that family history of diabetes and alcohol consumption were two of the predictors of severe periodontitis, increasing the probability of occurrence by 3.36 and 3.78, respectively. In model 2, PPD and CAL (mm) were significantly associated ( *p* < 0.001; *p* = 0.033) with the severity of periodontitis, increasing the probability of its occurrence by 3.64 and 4.71, respectively.

**Table 6:** Binary logistic regression analysis to estimate the association between different variables and severe periodontitis

Model and variables	Severe periodontitis (dichotomous)		
	OR	95% CI	*p* -Value
Model 1			
Family history of T1DM (yes vs. no)	3.36	1.22–9.21	0.019
Alcohol consumption (yes vs. no)	3.78	1.23–11.63	0.020
Model 2			
PPD total (mm)	3.64	14.74–90.34	< 0.001
CAL total (mm)	4.71	1.13–19.59	0.033

Abbreviations: CA, clinical attachment loss; CI, confidence interval; OR, odds ratio; PPD, periodontal probing depth; T1DM, type 1 diabetic mellitus.

Note: The *p* -value was derived from the one-way analysis of variance test and Bonferroni's test.

## Discussion

Despite the existence of numerous studies on the relationship between PD and Type 2 diabetes mellitus (T2DM), the relationship, particularly for T1DM, remains poorly studied.

The purpose of this cross-sectional study is to evaluate the role of metabolic control as a key factor leading to the development and severity of periodontitis and to compare periodontal and oral hygiene status with HbA1c.

DM is characterized by the presence of hyperglycemia that could lead to macro- and microvascular complications. 2 33 34 Previous studies showed the presence of characteristic microvascular changes in periodontal tissues associated with diabetic complications 10 20 35

The severity of periodontitis in diabetic subjects is greatly influenced by their metabolic state. 10 36 37 Thus, one of the main known risk factors for periodontitis is poor glycemic management. 5 Several studies showed that, even after controlling for confounding variables including age, gender, and oral hygiene, the risk of developing periodontitis rose thrice in individuals with diabetes compared with those without the disease. 23 36 38 The results of the present study evidenced that patients with T1DM and periodontitis had worse glycemic control when compared with nondiabetic ones, as indicated by higher concentrations of HbA1c. As a result, these patients exhibited increased BOP and PPD and increased CAL as compared with their healthy counterparts and controlled T1DM, which are important indicators of PD progression. These findings were in accordance with the study by Ajita et al 12 that assessed the frequency of PD in a group of 40 subjects (20 T1DM and 20 nondiabetics) aged between 18 and 50 years, in which all periodontal parameters were increased in systemically compromised individuals especially those who were poorly controlled, shown an increase in periodontal inflammation. This can be explained by the formation of advanced glycation end products (AGEs) in the periodontium, in response to the presence of hyperglycemia. The accumulation of AGEs and the interaction of AGEs with receptor for advanced glycation end products (RAGEs) contribute to osteoclastogenesis through an increase in the expression of the RANKL receptor activator and the downregulation of osteoprotegerin. The interaction between AGE–RAGE in monocytes activates the transcription factor nuclear factor-κB, which modifies the phenotype of monocytes/macrophages and results in increased production of proinflammatory cytokines that are at the root of periodontal destruction which can lead to tooth loss. 2 22 25

Another key important finding of this study was the number of tooth losses between the groups. Our findings showed that statistically significant differences were found between nondiabetic versus controlled diabetic subjects and between controlled versus uncontrolled diabetic individuals. These results are in agreement with the study by Popławska-Kita et al 1 which shows that uncontrolled T1DM patients exhibited a lower number of teeth when compared with controls, indicating that tooth loss due to periodontitis is closely influenced by the glycemic control. Another possible explanation for this augmented tooth loss in uncontrolled T1DM subjects is the release of reactive oxygen species (ROS). The invading bacteria trigger the release of inflammatory cytokines, leading to increases in the number and activity of neutrophils, which release ROS in periodontitis. These ROS, that might be increased by the formation of AGEs, can activate osteoclasts and promote osteoclast formation. 2 25

A logistic regression analyses by Kaur et al 39 revealed twofold higher odds for increased number of missing teeth for T1DM subjects in comparison with nondiabetic subjects after adjusting for confounders (OR = 1.93 [95% CI: 1.37, 2.71]. Age group stratification revealed that the association between T1DM and tooth loss was statistically significant in individuals aged 40 to 49 years (OR = 3.49 [95% CI: 1.92, 6.36]) and 50 to 59 years (OR = 4.54 [95% CI: 1.70, 12.10]). Furthermore, in a multivariable model by Chang et al, 40 adjusting for the variables such as fasting blood glucose level, showed that the number of missing teeth (≥15 teeth) remained positively associated with the occurrence of new-onset diabetes.

Oral hygiene habits were another aspect of this cross-sectional study. Even though 57.6% of uncontrolled diabetic patients reported brushing their teeth twice a day, only 19.2% reported using brushing aids, which compromises the effectiveness of toothbrushing. The role of microbial plaque in the etiology of PD is unquestionable. 2 These individuals are therefore predisposed to greater periodontal inflammation.

Lertpimonchai et al 11 conducted a systematic review and meta-analysis aiming to estimate the effects of oral hygiene on periodontitis and concluded that dental floss was not effective as a tool for removal of interdental plaque because it requires the user to be instructed about specific skills to be more effective. On the other hand, interdental brushes have been shown to be the most effective method for the removal of interdental plaque. According to our results, uncontrolled diabetic patients had a statistically higher PI compared with controlled and nondiabetic patients, which justifies the presence of a greater inflammatory response in this type of individuals. The presence of increased PI, in association with poor metabolic control, predisposes these subjects to a greater degree of periodontal destruction, as evidenced by the increased BOP, PPD, CAL, and the number of tooth loss. These findings are in agreement with the study by Ismail et al 20 showing that children with T1DM exhibited greater plaque accumulation than those without diabetes. In our study, PPD and CAL were significantly associated with the severity of periodontitis, increasing the likelihood of the disease by 3.64 and 4.71 times in type 1 diabetic patients.

Regarding the behavioral factors that modify or aggravate PD, we found that there is a higher prevalence of PD, especially in more advanced stages (III/IV), in older patients (≥46 years), which suggests that the expression of PD increases with age. Of these patients, 43.5% had a second level of schooling, which may explain their lack of knowledge regarding the oral hygiene habits and the use of brushing aids, as 73.9% of them did not use them, and how this habit influences plaque control and consequently a greater predisposition to inflammation. As we can see from the presence of increased PI and BOP values in these patients. In addition, many of these individuals may be unaware of the importance of good glycemic control and how this may be related to the risk of PD.

Furthermore, the results of this study showed that family history and alcohol consumption increase the risk of severe periodontitis by 3.36 and 3.78 times.

Therefore, oral health education, diagnosis, and treatment of PD should be recommended as early as possible by health professionals, especially in T1DM individuals.

In addition to hyperglycemia, diabetes complications can also be caused by an imbalance in lipid metabolism characterized by increased serum levels of low-density lipoproteins, TG, and fatty acids. 37 41 The presence of hyperlipidemia has a major role in the pathogenesis of diabetes and PD. 37 42

Some studies showed an increase in the plasma lipid profile, namely, total cholesterol (T-chol), TG, and low-density lipoprotein cholesterol in periodontally compromised patients. 43 However, according to our results, only statistically significant differences were found between patients with periodontitis stage III/IV and healthy periodontium ( *p* = 0.041) in relation to TG levels. These results can be explained by the presence of circulating monocytes exposed to TG, promoting the release of proinflammatory cytokines such as IL-1β,TNF-α, and a decrease in growth factors which in turn will promote chronic inflammation, progressive destruction of periodontal tissues, and a reduced capacity for repair. 35 41

There is controversy over the results of various studies in which concerns the importance of the duration of DM for the appearance of PD in individuals with diabetes. Some studies showed a correlation between the duration of diabetes and the severity of PD. Thus, patients with a longer disease history exhibited increased probing pocket depth, BOP, and clinical attachment levels. 12 44 In a study performed by Pranckeviciene et al, 23 these periodontal parameters were associated with a mean number of missing teeth in T1DM subjects. According to our results, diabetic individuals with more advanced stages of periodontitis III/IV showed a longer duration of the disease compared with those with periodontitis stage I/II and to their healthy counterparts. However, it is important to emphasize that there were no statistically significant differences between the groups.

The limitation of this study was the sample size which was limited to a specific geographic area that could be considered not representative of the whole population. In future, longitudinal observation is needed to determine whether HbA1c levels increase in response to an increase in periodontal parameters.

## Conclusion

The results of this study suggest that high HbA1c levels were positively related with periodontal status. Uncontrolled T1DM showed increased PI compared with controlled and nondiabetic patients, which justifies the presence of a greater inflammatory response in this type of individuals. PPD and CAL were significantly associated with the severity of periodontitis, increasing the likelihood of the disease by 3.64 and 4.71 times in type 1 diabetic patients. Additionally, the risk of severe periodontitis is increased by 3.36 and 3.78 times by alcohol consumption and family history, respectively.
